# The Foreign Journals: Anatomy and Physiology

**Published:** 1839-01

**Authors:** 


### PART THIRD.

### Selections from the British and Foreign Journals.

### I. THE FOREIGN JOURNALS.

### ANATOMY AND PHYSIOLOGY.

*On the Structure and Functions of tho Cerebral Nerves of the Frog.*
By Professor VOLKMANN, of Dorpat.

[THIS article notices not only the origin and functions of the cerebral nerves, but describes likewise in great detail their course, and the muscles which they supply. Our extracts shall be confined to the first two heads.]

I. *On the Origin of the Nerves.* Only eight separate nerves arise from the brain of the frog; as the facial, glossopharyngeal, accessory, and hypoglossus are supplied by branches from other nerves. The facial is a branch of the auditory; the ninth and eleventh pairs are contained in the tenth; and the first cervical nerve supplies the place of the hypoglossus. The motor oculi arises from the crus cerebri behind the tuber cinereum to which the pituitary gland is attached. The nerve runs outwards and forwards and passes behind the optic nerve through a cartilaginous plate, which represents the great wing of the sphenoid bone. The pathetic arises from the posterior and upper border of the corpora quadrigemina, passes outwards and downwards, and traverses the cartilaginous plate of the sphenoid, a little way above the foramen of the motor oculi. The trigeminus arises from the external border of the medulla oblongata, it runs forwards and outwards, and traverses the bony portion of the sphenoid, forming, in the foramen through which it passes, a reddish ganglion, which receives several nerves. The abducens takes its origin from the anterior fissure of the medulla oblongata and ends in the ganglion of the trigeminus. The auditory nerve arises immediately posterior to the abducens, and soon divides into two branches, one of which passes into the ganglion of the fifth. The pneumogastric nerve arises from the outer edge of the medulla oblongata, posterior to the auditory nerve, and receives some nervous fibres which appear to represent the glossopharyngeal. It is not joined by any fibres which can be supposed to represent the accessory of Willis.

II. *On the Functions of the Nerves.* The motor oculi, judging from its course, is a mixed nerve, and endows the muscles which it supplies alike with motion and sensation. It is distributed to all the muscles of the eye except two, and as a voluntary muscle cannot be supposed to be without sensation, seeing that this quality regulates the degree and direction of the motion, it must in consequence be admitted that the motor nerve of the muscles of the eyeball contains also sensory fibres. On irritating the motor oculi of a recently killed frog, within the cavity of the skull a variety of motions were induced; the eyeball was turned in various directions, and rolled as if by the action of the inferior oblique. On preparing the parts so as to bring several of the muscles into view, and again irritating the nerve, spasmodic twitches were observed in several of the recti, but no effect was produced in the superior oblique, in the external rectus, or in the suspensorius muscle.

The pathetic nerve of the frog, regarded in an anatomical point of view, must be considered as purely motor. On irritating the root of the nerve a motion of the eyeball is produced, which from its nature must be dependent on the action of the superior oblique; and when the parts are prepared so as to show the muscles, and the nerve is then irritated, the convulsions are seen confined to that muscle.

Division of the pathetic, before it unites with the nasal nerve, does not seem to cause the animal pain, but it should be remembered that frogs when tormented occasionally suppress the expression of pain. It is more difficult to determine by experiment the function of the sixth pair, as its fibres traverse the ganglion of the trigeminus. On irritating its root the eyeball was drawn violently inwards, and was covered by the membrana nictitans, or in some instances it was turned backwards. On displaying the muscles of the eye and then applying irritation to the nerve, contraction of the fibres of the rectus externus and suspensorius was distinctly observed; and on dividing, in another frog, the third and fourth pairs, and irritating the medulla oblongata with a needle, the eyes were violently drawn back into their sockets, and covered by the membrana nictitans. They remained shut a considerable time, and then suddenly opened, the eyeballs being drawn forwards and the membrana nictitans downwards. The irritation reapplied reproduced the above phenomena. In another frog the sixth pair of both sides was divided, and the motor oculi and the pathetic were then irritated. The eyeballs were in consequence slightly moved, but they were never withdrawn into their sockets, nor were they covered by the membrana nictitans. Dr. Volkmann therefore considers the abducens as the most important motor nerve of the eyes; it is much thinner than the motor oculi, but this depends upon the latter being a mixed nerve and including sensory fibres, whilst the former is purely motor.

The trigeminus is a mixed nerve, and supplies both integument and muscle. Irritation of its root produces contraction of the mental and temporal, of the mylohyoid and nasal muscles, but no motion of the muscles of the eyeball.

On irritating the facial nerve within the cavity of the skull, convulsions of the vertebro-maxillaris and tympano-maxillaris were produced, and on applying the stimulus of galvanism, not only these muscles were affected, but also the stylohyoideus anterior, and in the male the muscular sac of the larynx, or in the female the slender muscular fibres of the pharynx.

The nervus vagus is a mixed nerve, formed from the union of the ninth, tenth, and eleventh pairs. Irritation of its root within the cavity of the skull produced convulsions in the levator scapulæ inferioris, the stylohyoideus posterior, the stylopharyngeus, and the muscles of the larynx. The glossopharyngeal branch was divided, but irritation of its extremity produced no convulsions in the muscles which it supplies; it is therefore considered as purely a sensory nerve. The ramus recurrens is a motor branch; irritation of it caused convulsions in various parts of the larynx; in particular the glottis was drawn to the side of the irritated nerve, and would unquestionably have opened had the opposite side been fixed and not yielded mechanically to the contraction of the irritated muscles. The influence of the vagus upon the motions of the heart is very peculiar. The brain and spinal cord of a frog were destroyed, and the anterior extremities and sternum carefully removed, so as to expose the trunk of the nerve. About a quarter of an hour after the death of the animal, galvanic stimulus was applied to the vagus by means of eight pairs of four inch square plates, at the same time constantly breaking and reconnecting the chain. Immediately before the experiment, the heart was beating thirty strokes a minute; in the second minute after application of the stimulus, it beat thirty-three strokes, and continued to do so during the third, fourth, and fifth minutes, when the experiment was interrupted. Three quarters of an hour after death the heart was beating twenty-nine strokes a minute; the galvanic stimulus was reapplied and in the second minute afterwards it beat only eleven strokes, in the third thirty-one, and in the fourth thirty-four. The small number of strokes during the second minute appeared to be owing to intermission. The experiment was repeated two hours after death, when the heart was beating twenty-nine strokes in the minute. During the second minute after application of the galvanism the number of beats was twenty-six, and during the third only sixteen. The difference was owing to intermission; the strokes followed at regular intervals till an intermission of perhaps nearly half a minute occurred, and then they again followed in regular succession. Irritation of the vagus likewise augmented the peristaltic motions of the stomach and bowels. The first cervical nerve is also a mixed nerve, and irritation of its root produces convulsions in the muscles which it supplies.

[The above sketch is necessarily very imperfect, as we have been obliged to omit many anatomical details, the knowledge of which would have removed several obscurities. For their solution we must refer the reader to the original paper.]

*Müller's Archiv.* 1838. Heft i.

---

*Anatomical Researches into the Comparative Structure of the Cutaneous and Mucous Membrane.* By M. FLOURENS.

IN the skin of the European, the dermis is covered by two layers of epidermis, the one internal and the other external; in the black or coloured races there is, beneath these two membranes, an organ for the secretion of colouring matter. In the tongue, whether of man or of quadrupeds, there exists between the dermis and epidermis a peculiar body, called mucous body c orpus mucosum.) This body which appeared to Malpighi, who obtained it by boiling, to be reticulated, is proved by the more exact process of maceration, to be continuous and membranous; of the two layers of epidermis in the skin of the European, the internal is the most coloured; in the tongue the mucous body is the seat of all partial discolorations. The mamma, in the human species, is surrounded by an areola or circle of a lighter or darker brown, or bister-colour. This colour is proved by careful maceration to be due to the internal epidermis, which is of a deep brown; but when seen through the external layer has a light grayish aspect. In the skin, then, of the European, the second or internal epidermis is the seat of colour. In every part of the body this membrane is more coloured than the external layer, it is the seat, as we have seen, of the discoloration of the nipple; it is upon this too that the sun produces its tanning effect. The dermis is the seat only of freckles, and other minute discolorations. The tongue may be taken as the type of an entire group of mucous membranes; externally it is covered by the epidermis, beneath the epidermis lies the mucous body, and beneath this again the dermis with its papillæ. The mucous body which exists in the tongue and which is found also on the palate, cheeks, and throughout the entire cavity of the mouth, extends still farther throughout the œsophagus. At the point where the œsophagus terminates and the stomach begins, an entirely different structure, which will form a subject for future enquiry, commences.

The characters of the mucous body are everywhere the same. In man it is white, in the ox it is the seat of those partial discolorations which are so often seen upon the palate and tongue of that animal. Its tissue is peculiar; it is rendered more compact, and, when its colour is white, becomes more white by boiling. It consists of layers superimposed and adherent. The second or internal epidermis is very thin and fine; it is covered in the areola of the nipple by a more or less distinct layer of pigmentum, and readily passes to a diffluent state. There is no doubt that the fable of a mucous membrane belonging to the skin took its rise from this state of the internal epidermis. This membrane can be obtained only by a certain degree of maceration; if the process is not carried on long enough it is detached with the external epidermis, if it is pushed too far, the membrane is dissolved away. Between these two degrees of maceration there is a point at which the internal epidermis is separated as a continuous and distinct layer. Those anatomists who have not continued the maceration long enough have denied the existence of a mucous membrane; those who have pushed the process too far have spoken of a mucous body, of a species of mucosity, or of a mucous and gelatinous liquid, (Meckel). Maceration, properly conducted, demonstrates, in place of this mucosity, a *bonâ fide* membrane, thin, and coloured—the internal epidermis.

The internal epidermis and the mucous body then are two distinct tissues, the former being to the skin what the latter is to the group of mucous membranes of which we are speaking. It is important to determine the precise point at which the one terminates and the other begins. If we examine the lips carefully, we trace a distinct continuity, on the one hand, between the external epidermis of the skin and the epidermis of the mucous membrane; on the other, between the dermis of the skin and that of the mucous membrane; but at the line which separates the pale from the coloured part of the lip, the mucous body takes the place of the internal epidermis.

The mucous membrane which covers the tongue extends, in the ox, over the entire cavity of the mouth, throughout the œsophagus, and over the three first stomachs; in the horse, it terminates in the stomach itself. The structure of this membrane varies somewhat in the several parts which it covers. It is thinner on the cheeks than on the tongue. Near the lips it is furnished with numerous long papillæ. Each of these papillæ is surrounded by two sheaths, the one furnished by the epidermis, the other by the mucous body. The same structure prevails in the palate, the dermis of which is arranged in transverse lines bristling with papillæ. The mucous body, which is the seat of all discolorations, is composed of layers superimposed and adherent, and these layers again consist of perpendicular *blades.* The mucous membrane of the œsophagus and of the three first stomachs of the ox closely resembles that of the tongue and mouth. The papillæ, though differing in shape and arrangement in the several parts, are everywhere covered by two sheaths derived from the mucous body and from the epidermis. After sufficient maceration these sheaths may be detached from the papillæ like the fingers of a glove. The mucous membrane of the horse differs but little from that of the ox. On the palate we observe the same transverse lines, but without papillæ. It covers a part of the stomach and exactly resembles the lining membrane of the three first stomachs of the ruminantia.

In the horse, then, as well as in the ox, there exists an entire group of mucous membranes precisely similar to the membrane covering the tongue. In the one, as in the other, this membrane lines the entire cavity of the mouth. In the horse, it extends also through the œsophagus and first part of the stomach, in the ox through the œsophagus and three first stomachs.

*Gaz. Méd. de Paris. Mars,* 1838.

---

*On the Cicatrization of Tendons.* By M. Bouvier.

M. Bouvier has been lately engaged in making experiments on this subject; and he exhibited some of the results at a recent meeting of the French Academy. His experiments went to ascertain, 1, how far the divided ends of a tendon may be separated from one another, immediately after their division, so as not to prevent their reunion by an intermediate tissue; 2, in what degree the new tissue approaches to that of true tendinous matter. The tendinous cicatrices presented to the Academy were taken, 1, from a horse, two months before it was killed, and consisted of a perforating tendon of one of the anterior extremities; 2, from a dog, which was killed six months after the division of the extensor tendon of the foot. Each of these cicatrices was about two inches and a half in length. That of the horse consisted of a grayish, compact tissue, consisting of irregular fibres not very conspicuous, contrasting with the pearly appearance of the true tendon. The cicatrix of the dog consisted of longitudinal fibres, more delicate than those of the tendon, and arranged differently, being also very distinct from these.

*Bulletin de l'Academie de Médecine.* No. xvi. 1838

---

*On the Venous Circle of the Mammary Areola.* By Professor Sebastian.

In dissecting the mammæ, Professor Sebastian had frequently observed a filament beneath the areola, apparently describing a circle round it; but being unable to procure the gland of a woman giving suck, he for a long while deferred the investigation of its nature. However, by boiling an empty mammæ for twenty-four hours, the close cellular tissue of the organ was so effectually loosened, that an excellent substitute for the full gland was obtained. By examining it he satisfied himself that underneath the skin of the female areola a circle exists, which usually surrounds the greatest part of the base of the nipple at the distance of a line and a half from it. In some cases instead of being circular it is angular, its angles giving origin to branches running towards the circumference of the areola; other smaller twigs ascend from it into the nipple itself. Its vascular and venous nature was proved by injection. The circle exists in the male also, though in him it exhibits a somewhat different form. This anatomical fact has altogether escaped the notice of modern observers, at least no mention is made of it by Meckel, Cloquet, Weber, Lenhossek, &c. The indefatigable Haller, however, distinctly described it in his Elements of Physiology, vol. vii. sect. 1. Sebastian proposes in consequence that it be called Haller's circle. As to its use, he believes that it has much to do with the erection of the nipple. Hitherto that part of the breast has been referred to the class of erectile tissues, more on account of its exhibiting the phenomenon of erection, than from anatomical demonstration of its structure. But when the venous circle becomes turgid from being filled with blood, and at the same time the veinules forming communications between it and the nipple are filled, the whole apparatus must push up and cause the erection of the nipple.

*Tijdschrift voor Natuurlijke Geschiedenis.* 11 Deel., 3 Stuk.

---

*On accessory Supra-Renal Capsules.* By Professor SEBASTIAN.

IN the body of a woman who died of general dropsy, with tubercular disorganization of the kidney, I discovered, attached to one of the supra-renal capsules, corpuscula of a different shape from that of the capsule itself, not more than a line and a half broad, but of the same colour and structure as that organ. There were evident fibres in the cortical substance and internally a distinct cavity. These characters justify me in considering the bodies described as supernumerary capsules. They could scarcely be looked on as lobes of the principal gland, as they were only united to it by loose cellular membrane. I never felt persuaded of the close relation of the supra-renal capsules to the lymphatic, but have always felt inclined to refer its function to the vascular system. To me the vein issuing from it appears to fill the office of an excretory duct, and to convey either a material secreted from the arterial blood, or that fluid itself modified in its properties, and destined for the improvement of the venous blood. The great size of the organs in the fœtus is thus accounted for, as also the peculiar disposition of the vein itself, which is such that by it the whole gland is easily distended. Thus too is explained the fact, that in diseases of the venous system these glands are not unfrequently found either increased in bulk or otherwise unhealthy. According to this view, therefore, the capsules would act the part of a placenta. I have not discovered any distinction between the globules of the supra-renal and renal veins.

*Tydsch. voor Natuurl. Gesch.* 3 *Deel.*

